# Investigation of abdominal artery delineation by photon-counting detector CT

**DOI:** 10.1007/s11547-024-01858-z

**Published:** 2024-07-24

**Authors:** Takashi Ota, Hiromitsu Onishi, Toshihide Itoh, Hideyuki Fukui, Takahiro Tsuboyama, Atsushi Nakamoto, Yukihiro Enchi, Mitsuaki Tatsumi, Noriyuki Tomiyama

**Affiliations:** 1 Department of Diagnostic and Interventional Radiology, Osaka University Graduate School of Medicine, D1, 2-2, Yamadaoka, Suita, Osaka 565-0871 Japan; 2 Department of CT Research and Collaboration, Siemens Healthineers, Tokyo, Japan; 3 Department of Medical Technology, Osaka University Hospital, Suita, Japan

**Keywords:** Computed tomography angiography, Three-dimensional imaging, Hepatic artery, Superior mesenteric artery, Renal artery

## Abstract

**Objectives:**

To evaluate the ability of 50-keV virtual monoenergetic images (VMI) to depict abdominal arteries in abdominal CT angiography (CTA) compared with 70-keV VMI with photon-counting detector CT (PCD-CT).

**Methods:**

Fifty consecutive patients who underwent multiphase abdominal scans between March and April 2023 were included. Signal-to-noise ratio (SNR) and contrast-to-noise ratio (CNR) were quantitatively assessed for the abdominal aorta (AA), celiac artery (CeA), superior mesenteric artery (SMA), renal artery (RA), and right hepatic artery (RHA) at both 50- and 70-keV VMI. In addition, 3D images from CTA were analyzed to measure arterial lengths and evaluate the visualization of distal branches.

**Results:**

Significantly higher SNR and CNR were observed at 50-keV compared to 70-keV VMI for all arteries: AA (36.54 and 48.28 vs. 25.70 and 28.46), CeA (22.39 and 48.38 vs. 19.09 and 29.15), SMA (23.34 and 49.34 vs. 19.67 and 29.71), RA (22.88 and 48.84 vs. 20.15 and 29.41), and RHA (14.38 and 44.41 vs. 13.45 and 27.18), all *p* < 0.05. Arterial lengths were also significantly longer at 50-keV: RHA (192.6 vs. 180.3 mm), SMA (230.9 vs. 216.5 mm), and RA (95.9 vs. 92.0 mm), all *p* < 0.001.

**Conclusion:**

In abdominal CTA with PCD-CT, 50-keV VMI demonstrated superior quantitative image quality compared to 70-keV VMI. In addition, 50-keV VMI 3D CTA allowed better visualization of abdominal artery branches, highlighting its potential clinical advantage for improved imaging and detailed assessment of abdominal arteries.

## Introduction

Abdominal CT angiography (CTA) is a widely used, noninvasive method for evaluating not only abdominal arterial anatomy but also vascular disorders [1, 2]. Preoperative knowledge of arterial anatomy and variants is crucial for treatment selection, surgical planning, and iatrogenic injury avoidance, particularly in patients with pancreatic, hepatobiliary, and renal malignancies [3, 4]. The image quality of abdominal vessels has significantly improved with the introduction of multidetector-row CT and ultra-high-resolution CT (UHRCT) scanners, which have specifically improved the visibility of small arteries [5, 6]. This technological progress has enabled precise and consistent visualization of microvascular anatomy, thereby presenting essential information for preoperative planning of microsurgery [7, 8]. To date, there is a lack of quantitative research investigating the extent to which advancements in CTA imaging technology have facilitated the accurate identification of small arteries.

To date, many studies have been conducted to improve the image quality of CTA images. Recently, UHRCT was introduced, which has more than doubled spatial resolution [9]. UHRCT is good at showing small arteries, such as small abdominal visceral arteries [6], renal artery (RA) in-stent stenosis [10], peripheral arteries of the lower extremities [9], and Adamkiewicz artery [11]. Another approach is to increase the contrast of vessels using low-keV virtual monoenergetic images (VMI) with dual-energy CT (DECT). By varying the energy level of the VMI, different contrasts can be achieved, with lower energies primarily increasing the contrast effect of iodine and improving the image contrast [12, 13]. Low-keV VMI below 60-keV has been shown to improve the contrast-to-noise ratio (CNR) and qualitative image quality [14–16]. In addition, the use of low-keV VMI in DECT produces contrast enhancements comparable to those in conventional CT images, even when the contrast media are reduced [17].

Photon-counting detector CT (PCD-CT) is a new technology that overcomes the limitations of conventional CT by using photon-counting detectors [18, 19]. PCD-CT generates a VMI by utilizing the inherent spectral data acquired by the photon-counting detectors. These detectors can directly quantify individual photons and differentiate between photons with varying energies. The ability to resolve energy enables the collection of spectral CT data across multiple energy bins [20]. Energy bins in PCD-CT are essential for the generation of VMI and are determined by energy thresholds that specify the range of photon energies counted within each bin [21]. PCD-CT can improve the image quality of VMI at low-keV levels because the spatial resolution is improved and the image noise of PCD-CT is lower than that of DECT [18, 19].

In this study, we hypothesized that abdominal artery delineation during PCD-CT is enhanced by low-keV VMI. The term “abdominal artery delineation” refers to the process of outlining or marking the boundaries of abdominal arteries. This is a crucial step in medical imaging and surgical planning because it allows for the accurate identification and assessment of these vital structures [22]. Therefore, we quantitatively determined the extent to which the abdominal arteries could be delineated in the 3D CTA images by automatically measuring the length of the arteries using a dedicated application and comparing the artery length. This retrospective study evaluated the ability of low-keV (50-keV) VMI to depict abdominal arteries compared to 70-keV VMI using PCD-CT.

## Materials and methods

### Study design and patient population

**Fig. 1:**
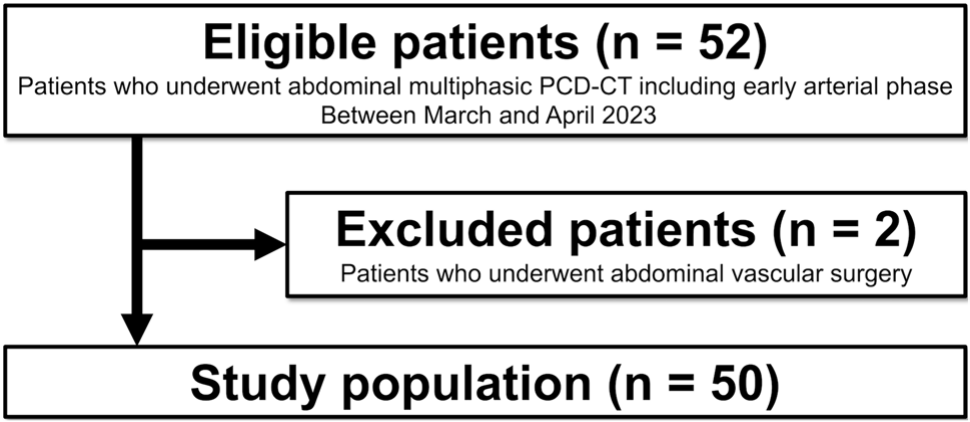
Flowchart of patient enrollment

This single-center retrospective study was conducted at a university medical hospital and was approved by the institutional review board and local ethics committee. The requirement for informed consent was waived because this was a retrospective, non-interventional, observational study. Consecutive patients who underwent multiphasic abdominal PCD-CT including the early arterial phase were enrolled between March and April 2023. The exclusion criteria were patients who had undergone abdominal vascular surgery were excluded (Fig. 1).

### CT scan protocol

All CT images were acquired using a clinical dual-source PCD-CT scanner (NAEOTOM Alpha, Siemens Healthineers, Forchheim, Germany). All scans were acquired in single-source, multi-energy mode (Quantum Plus) using a tube voltage of 120 kV, collimation of 120 × 0.2 mm, pitch factor of 0.8, and gantry rotation time of 0.50 s. The VMI energy levels were 50 and 70-keV. The low-keV energy was set to 50-keV because it has been reported that 50-keV VMI has the best image quality in PCD-CT for abdominal CTA [23, 24]. As recommended by the manufacturer, the tube current–time product was set to an image quality level of 170, and automatic tube current modulation (CARE Dose4D; Siemens Healthineers) was used. The protocol for upper abdominal multiphasic CT imaging was 600 mgI per unit body weight (kg) of the nonionic iodine contrast material, iomeprol (Iomeron 350; Eisai, Tokyo, Japan), which was administered intravenously to the patient in 25 s. An early arterial phase scan was initiated with bolus tracking of the abdominal aorta (AA) with a threshold of 40 HU and a delay of 8 s. Because a 70-keV VMI exhibits approximately the same contrast as an image with a tube voltage of 120 kV, which is commonly used in conventional CT, the 70-keV image was used as a standard [25].

### Image reconstruction

Early arterial phase images were reconstructed in the axial plane by quantum iterative reconstruction (QIR; Siemens Healthineers) at the highest intensity level of 4 using the vascular reconstruction kernel (Bv44). The field of view was 345 mm and the matrix size was 512 × 512. The thickness was 0.4 mm without a gap during the early arterial phase. The 3D volume rendering (VR) images of the abdominal artery were reconstructed from the early arterial phase VMI (50 and 70-keV) using a 3D image analysis system (SYNAPSE VINCENT Version 6.7; Fujifilm, Tokyo, Japan).

### Quantitative analysis

Two radiologists (both with 11 years of abdominal imaging experience) independently placed regions of interest (ROIs) in the following areas: AA, celiac artery (CeA), superior mesenteric artery (SMA), RA, and right hepatic artery (RHA) and measured the CT attenuation at 50 and 70-keV VMI reconstructed from early arterial phase CT. The mean CT and standard deviation (SD) values for each artery measured by the two readers were averaged. Image noise (SD_fat) was defined as the SD of CT density of the anterior subcutaneous fat. The mean CT value of the muscles (HU_muscle) was defined as the CT value of the erector spinae muscles at the same slice and level as the CeA. The signal-to-noise ratio (SNR) and CNR were calculated as follows:

$$ \begin{gathered} {\text{SNR}} = {\text{HU}}\_{\text{vessel}}/{\text{SD}}\_{\text{vessel}} \hfill \\ {\text{CNR}} = \left( {{\text{HU}}\_{\text{vessel}} - {\text{HU}}\_{\text{muscle}}} \right)/{\text{SD}}\_{\text{fat}}. \hfill \\ \end{gathered} $$

### Automatic length measurement of visualized abdominal arteries

One radiologist (with 11 years of experience in abdominal imaging and specializing in 3D image processing) measured the length of the artery by analyzing the 3D VR images using the following steps:

*Step 1* In the early arterial phase, VR images of the abdominal arteries were created at 50- and 70-keV VMI using SYNAPSE VINCENT. The observer was blinded to whether each CT image was a 50- or 70-keV CTA image.

*Step 2* For the VR images, an automatic bone removal function was used to remove the bone and extract the entire artery of the upper abdomen.

*Step 3* Cutting and vessel selection tools were used to manually extract the origin of the CeA to the periphery of the RHA, origin of the SMA to the periphery, and origin of the left RA to the periphery.

In cases in which the RHA bifurcated from the SMA (replaced RHA), we manually extracted VR images from the beginning of the SMA to the RHA. When extracting the renal artery, the opacity was adjusted to make the end of the renal artery visible, because the end of the renal artery was not visible at the default opacity because of the renal parenchyma. If an accessory RA was present, extraction was performed from the main RA to the periphery.

*Step 4* In the vessels extracted in Step 3, the vessel pathways were automatically extracted using SYNAPSE VINCENT curved planar reformation (CPR) analysis.

*Step 5* For the RHA measurements, the pathway from the CeA origin to the A8 periphery was selected from the vessel pathways, and the distance was measured automatically. For SMA measurements, the pathway was extracted using CPR analysis. The pathway from the origin of the SMA to the end of the ileocolic artery (ICA) was selected, and the distance was measured automatically. To measure the length of the RA, the path from the origin of the left RA to the periphery of the anterior superior segmental artery (ASSA) was selected and the distance was measured automatically.

**Fig. 2:**
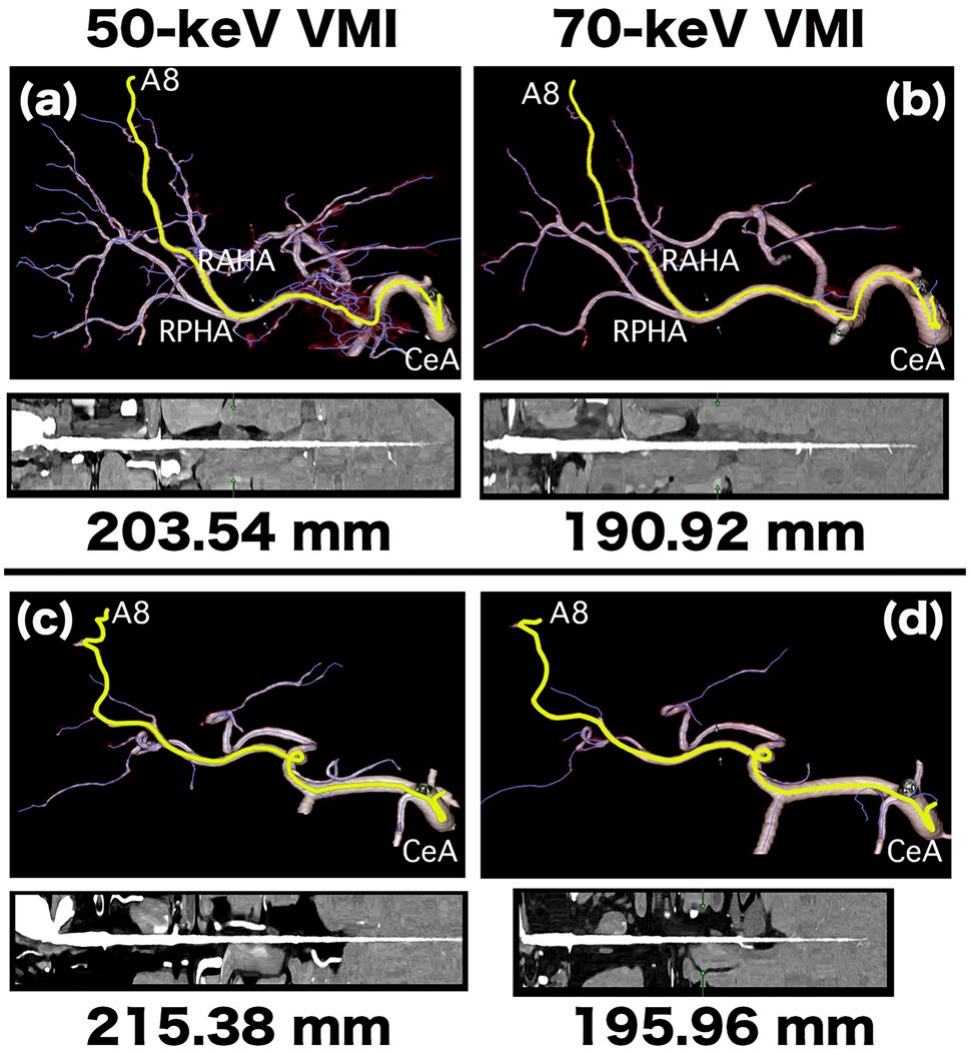
3D images of a 60-year-old woman (**a, b**) and a 79-year-old man (**c, d**) for right hepatic artery (RHA) length measurement. (**a, b**) We performed curved planar reformation (CPR) analysis on cropped volume rendering (VR) images to extract vascular pathways on 50- and 70-keV virtual monoenergetic images (VMI). The purple line indicates that the software automatically extracted the vascular pathway and the yellow line indicates the pathway from the CeA origin to the A8 periphery. The results of the artery length measurements are shown in the bottom row. CeA, celiac artery; RAHA, right anterior hepatic artery; RPHA, right posterior hepatic artery. (**c, d**) We performed CPR analysis on the cropped VR images to extract the vascular pathways on 50- and 70-keV VMI. The results of the artery length measurements are displayed in the bottom row

**Fig. 3:**
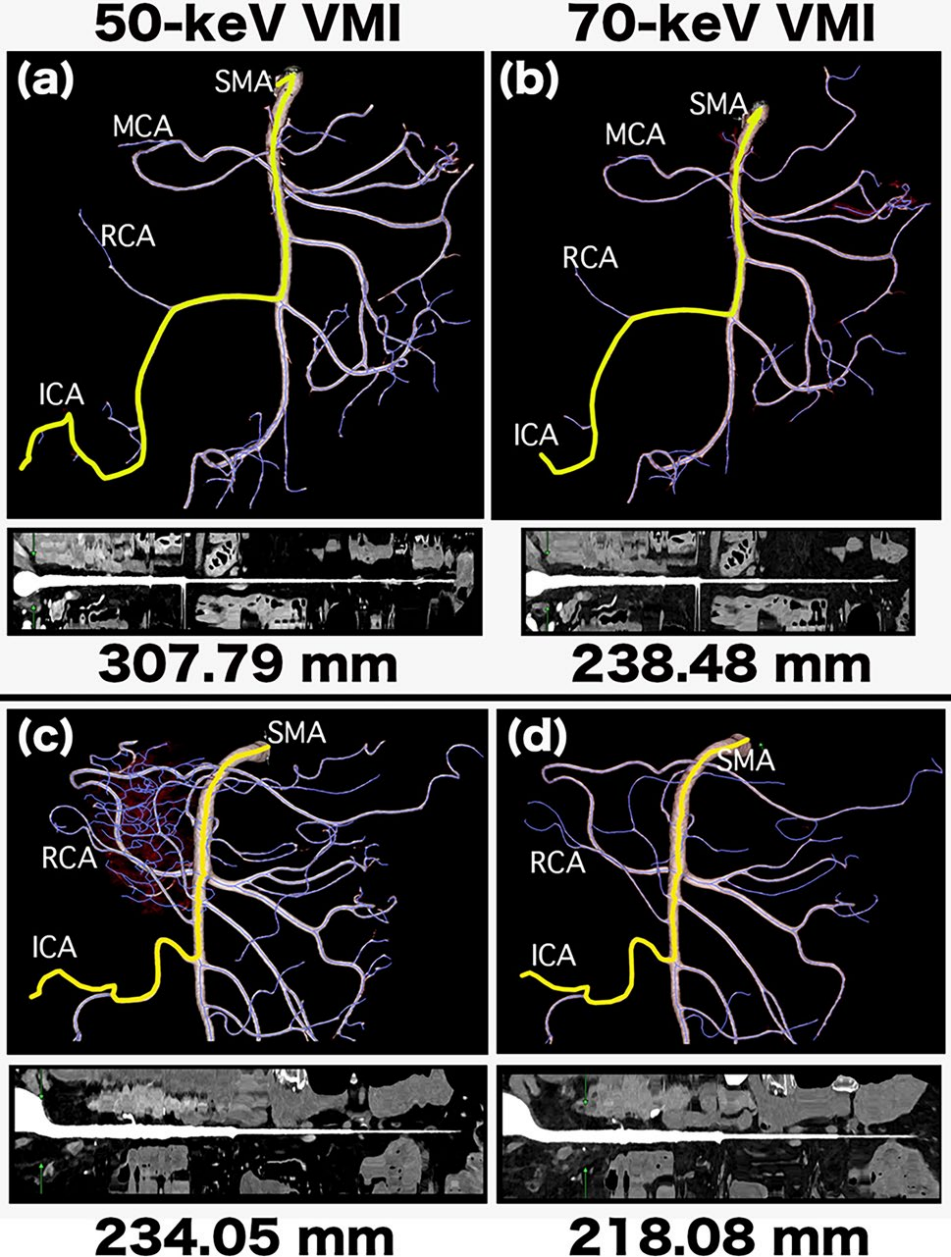
3D images of a 75-year-old woman (**a, b**) and a 68-year-old man (**c, d**) for superior mesenteric artery (SMA) length measurement. (**a, b**) We performed curved planar reformation (CPR) analysis on the cropped volume rendering (VR) images to extract the vascular pathways on 50- and 70-keV virtual monoenergetic images (VMI). The purple line indicates that the software automatically extracted the vascular pathway, and the yellow line indicates the pathway from the SMA origin to the ileocolic artery (ICA) periphery. The results of the artery length measurements are displayed in the bottom row. MCA, middle colic artery; RCA, right colic artery. (**c, d**) CPR analysis was performed on the cropped VR images to extract the vascular pathways on 50- and 70-keV VMI. The results of the artery length measurements are displayed in the bottom row

**Fig. 4:**
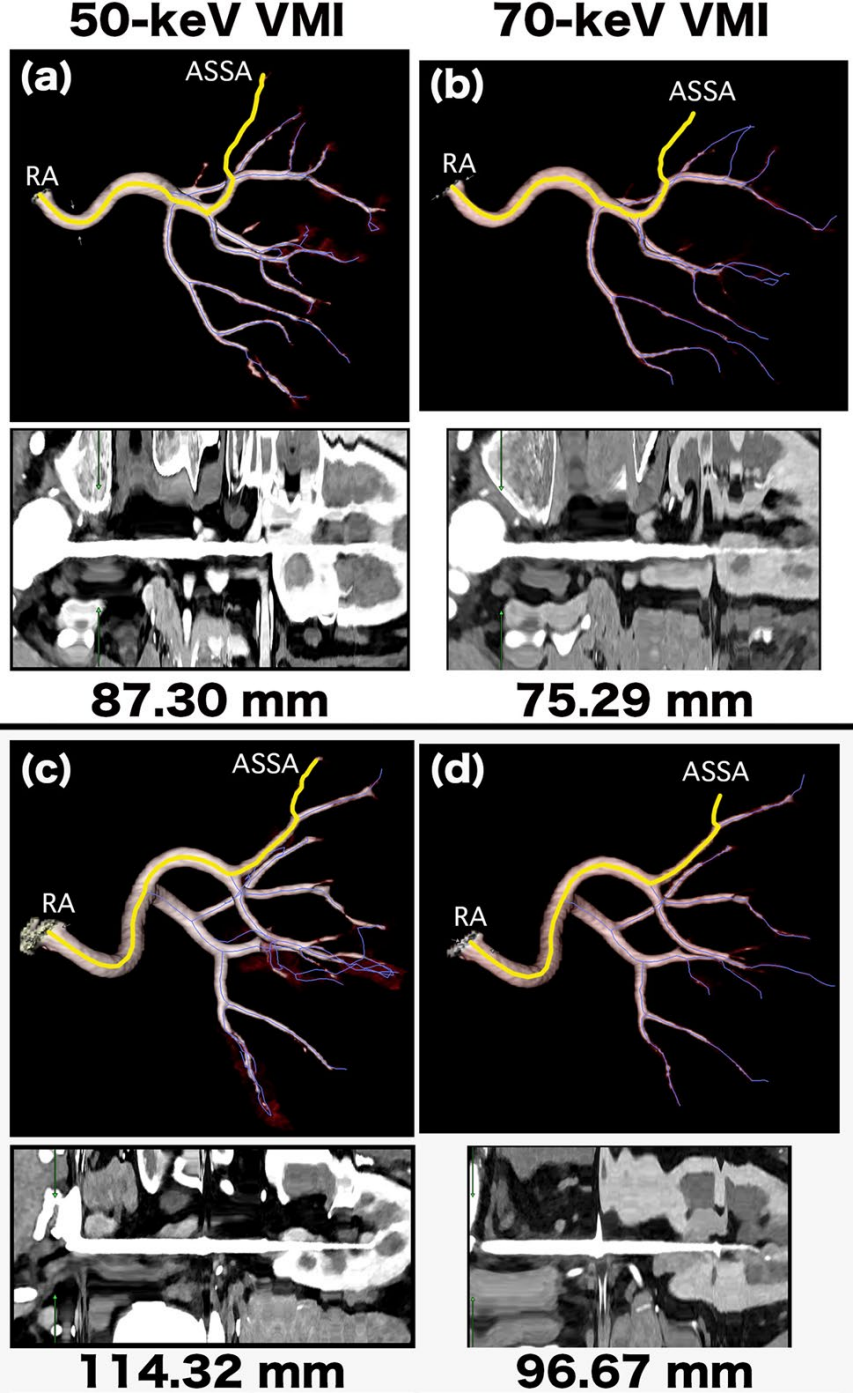
3D images of a 61-year-old woman (**a, b**) and a 68-year-old man (**c, d**) for renal artery (RA) length measurement. (**a, b**) We performed curved planar reformation (CPR) analysis on the cropped volume rendering (VR) images to extract the vascular pathways on 50- and 70-keV virtual monoenergetic images (VMI). The purple line indicates that the software automatically extracted the vascular pathway, and the yellow line indicates the pathway from the RA origin to the anterior superior segment artery (ASSA) periphery. The results of the artery length measurements are displayed in the bottom row. (**c, d)** We performed CPR analysis on the cropped VR images to extract the vascular pathways on 50- and 70-keV VMI. The results of the artery length measurements are displayed in the bottom row.

3D images of the RHA, SMA, and RA length measurement methods are summarized in Figs. 2, 3, and 4, respectively. To determine which artery exhibited the greatest improvement in delineation at low-keV, we also calculated and compared the difference in the length of the RHA, SMA, and RA between 50 and 70-keV VMI.

### Statistical analysis

Because all parameters were normally distributed using the Shapiro–Wilk test, parametric tests were performed. The SNR and CNR for each vessel at 50 and 70-keV VMI were compared using a paired *t*-test. The inter-reader reliability of the CT and SD values for each artery between the two observers was assessed by calculating intraclass correlation coefficients (ICCs) [26]. The lengths of each artery at 50 and 70-keV VMI were also compared using a paired t-test. The difference in arterial length at 50 and 70-keV VMI was compared between the three groups (CeA, SMA, and RA) using one-way analysis of variance (ANOVA) with post-hoc Tukey’s test. SPSS for Mac version 24 (IBM, Chicago, USA) was used for all statistical analyses. *p* values < 0.05 were considered to indicate a statistically significant difference.

## Results

### Patient characteristics

During the study period, 52 patients underwent PCD-CT for initial examination of tumors in the upper abdomen. Two patients were excluded because they underwent open abdominal vascular repair. The final study population included 50 patients (mean ± SD age: 64.5 ± 14.2 years; range: 26–82 years); specifically, 28 men and 22 women (Fig. 1, Table 1). The 50 patient characteristics are summarized in Table 1. The radiation dose parameters for the early arterial phase in this study were as follows: volume CT dose index, 8.26 ± 1.61 mGy; dose-length product, 237.30 ± 65.07 mGy•cm; effective dose, 3.56 ± 0.98 mSv (Table 1).

**Table 1:** Patient demographics

Characteristics	Value
No. of patients	**50**
Mean age (y)	**64.5 ± 14.2 (26–82)**
*Sex*	
No. of women	**22**
No. of men	**28**
Injection rate of contrast material (mL/s)	**3.92 ± 0.82 (2.1–5.3)**
*Radiation dose of early arterial phase*	
CTDI vol (mGy)	**8.26 ± 1.61 (5.4–11.9)**
DLP (mGy cm)	**237.30 ± 65.07 (120–410)**
Effective dose (mSv)	**3.56 ± 0.98 (1.8–6.2)**

CTDI vol, volume CT dose index; DLP, dose-length product

Numbers in parentheses indicate ranges

### Quantitative analysis

The ICCs of the two radiologists for each vessel are summarized in Table 2. The ICCs for the CT values between the two observers showed moderate-to-excellent reliability. The ICCs for the SD values between the two observers showed poor to good reliability (Table 2).

**Table 2:** Intraclass correlation coefficients between two radiologists for quantitative image analysis

	HU_muscle	SD_fat	HU_AA	SD_AA	HU_CeA	SD_CeA
*50-keV VMI*						
ICC	0.91	0.67	0.99	0.70	0.98	0.50
95% C.I	0.85–0.95	0.52–0.82	0.99–1.00	0.48–0.83	0.97–0.99	0.26–0.68
*70-keV VMI*						
ICC	0.90	0.79	0.99	0.64	0.98	0.57
95% C.I	0.83–0.94	0.66–0.87	0.99–1.00	0.45–0.78	0.97–0.99	0.35–0.73

	HU_SMA	SD_SMA	HU_RA	SD_RA	HU_RHA	SD_RHA
*50-keV VMI*						
ICC	0.98	0.46	0.98	0.57	0.64	0.42
95% C.I	0.97–0.99	0.21–0.66	0.96–0.99	0.31–0.75	0.44–0.78	0.16–0.63
*70-keV VMI*						
ICC	0.98	0.58	0.98	0.49	0.73	0.41
95% C.I	0.97–0.99	0.34–074	0.96–0.99	0.24–0.68	0.56–0.83	0.15–0.61

VMI, virtual monoenergetic image; ICC, intraclass correlation coefficient; 95% C.I., 95% confidence interval; HU, Hounsfield Unit; SD, standard deviation; AA, abdominal aorta; CeA, celiac artery; SMA, superior mesenteric artery; RA, renal artery; RHA, right hepatic artery

**Fig. 5:**
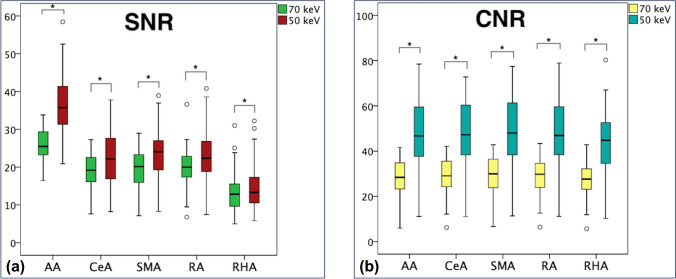
Box plots of signal-to-noise ratio (SNR) and contrast-to-noise ratio (CNR) of each artery. The centerline of the box plot is the median value, the box represents the interquartile range, and the whiskers represent the range of values. (**a**) Box plot of SNR of each artery. The green box plot shows the 70-keV VMI, and the red box plot shows the 50-keV VMI. The SNR of each artery was significantly higher at 50-keV than at 70-keV VMI (all *p* < .05). (**b**) Box plot of CNR for each artery. The yellow box plot shows the 70-keV VMI, and the blue box plot shows the 50-keV VMI. The CNR of each artery was significantly higher at 50-keV than at 70-keV VMI (all *p* < .001). AA, abdominal aorta; CeA, celiac artery; SMA, superior mesenteric artery; RA, renal artery; RHA, right hepatic artery. The asterisks indicate statistically significant differences

The SNR and CNR values for each vessel are listed in Table 3. The SNR values for the 50-keV VMI were significantly higher than those for the 70-keV VMI for each artery (all *p* < 0.05). The CNR values for 50-keV VMI were also significantly higher than those for 70-keV VMI for each artery (all *p* < 0.001) (Table 3, Fig. 5).

**Table 3:** The SNR and CNR of abdominal arteries

	AA	CeA	SMA	RA	RHA
*SNR*					
50-keV VMI	36.54 ± 7.62	22.39 ± 7.25	23.34 ± 7.08	22.88 ± 6.96	14.38 ± 5.89
70-keV VMI	25.70 ± 4.36	19.09 ± 4.59	19.67 ± 4.75	20.15 ± 5.00	13.45 ± 5.31
*p*-value	< 0.001	< 0.001	< 0.001	< 0.001	0.02
*CNR*					
50-keV VMI	48.28 ± 13.99	48.38 ± 13.47	49.34 ± 14.42	48.84 ± 14.01	44.41 ± 13.55
70-keV VMI	28.46 ± 7.44	29.15 ± 7.44	29.71 ± 7.84	29.41 ± 7.55	27.18 ± 7.01
*p*-value	< 0.001	< 0.001	< 0.001	< 0.001	< 0.001

SNR, signal-to-noise ratio; CNR, contrast-to-noise ratio; keV, kiloelectron volt; VMI, virtual monoenergetic imaging; AA, abdominal aorta; CeA, celiac artery; SMA, superior mesenteric artery; RA, renal artery; RHA, right hepatic artery

### Length of abdominal arteries

#### RHA, SMA, and RA length

**Fig. 6:**
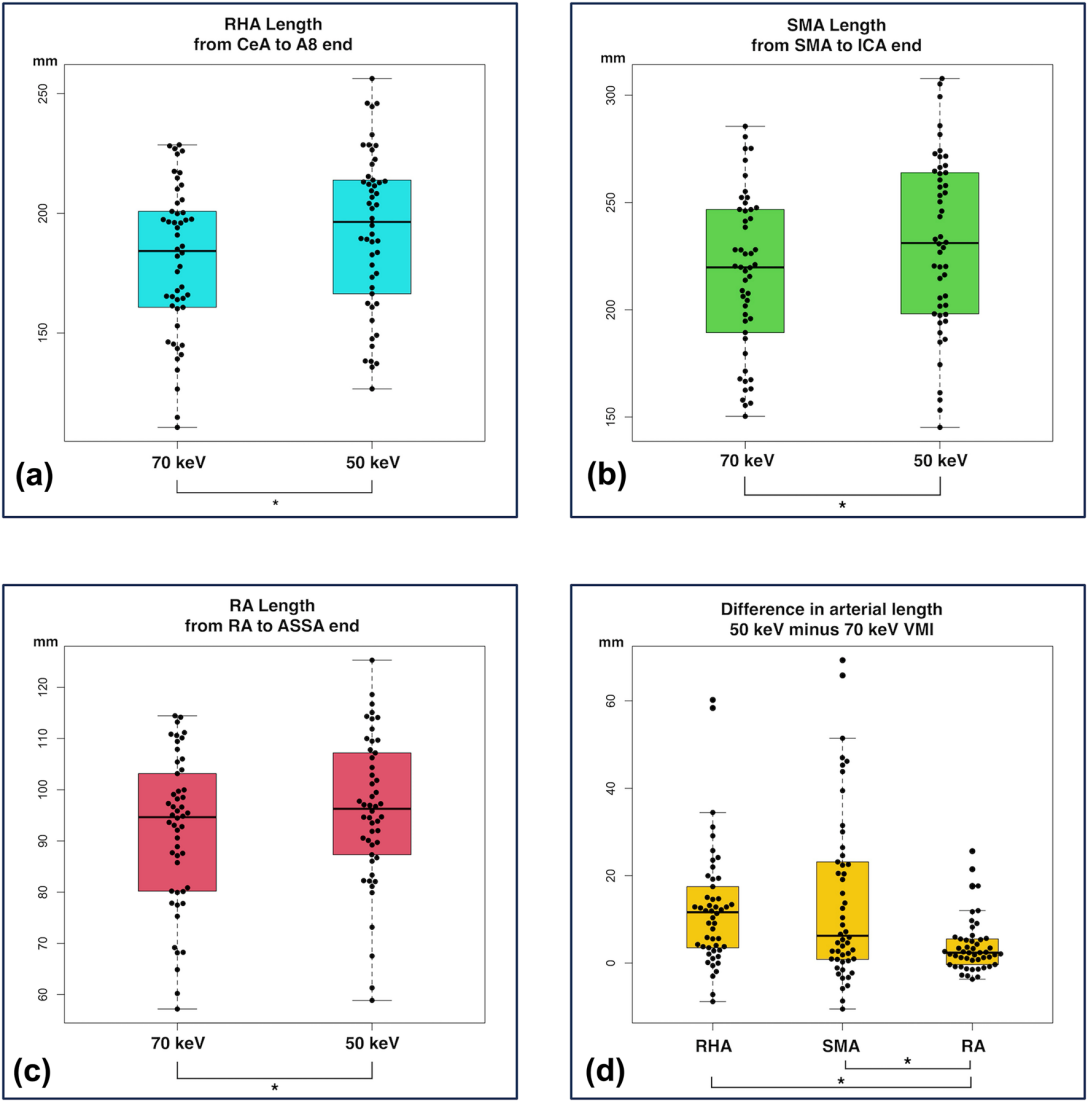
Box plots of arterial lengths at 50 and 70-keV VMI. (**a**) Right hepatic artery (RHA) length from the celiac artery (CeA) to the A8 end. The RHA length was significantly longer at 50-keV than at 70-keV VMI. (**b**) Superior mesenteric artery (SMA) length from SMA origin to ileocolic artery (ICA) end. The SMA length was significantly longer at 50-keV than at 70-keV VMI. (**c**) Renal artery (RA) length from RA origin to anterior superior segmental artery (ASSA) end. The RA length was significantly longer at 50-keV compared than that at 70-keV VMI. (**d**) The difference in arterial length between two energies were significantly longer in the RHA and SMA than in the RA. The asterisks indicate statistically significant differences

The distance between the CeA origin and the A8 end was significantly longer for 50-keV VMI than for 70-keV VMI: 192.6 ± 33.3 mm versus 180.3 ± 30.9 mm, respectively (*p* < 0.001). The distance between the SMA origin and the ICA end was significantly longer for 50-keV VMI than for 70-keV VMI: 230.9 ± 40.6 mm versus 216.5 ± 37.0 mm, respectively (*p* < 0.001). The distance between the left RA origin and the ASSA end was significantly longer for 50-keV VMI than for 70-keV VMI: 95.9 ± 14.4 mm and 92.0 ± 14.7 mm, respectively (*p* < 0.001) (Table 4, Fig. 6).

**Table 4:** The length of abdominal arteries and the difference in arterial length between 50 and 70-keV VMI

Artery length	RHA from CeA to A8	SMA from SMA to ICA	RA from RA to ASSA
50-keV VMI (mm)	192.6 ± 33.3	230.9 ± 40.6	95.9 ± 14.4
95% C.I. (mm)	183.2–202.1	219.3–242.4	91.8–100.0
Range (mm)	126.7–256.3	145.2–307.8	58.9–125.3
70-keV VMI (mm)	180.3 ± 30.9	216.5 ± 37.0	92.0 ± 14.7
95% C.I. (mm)	171.5–189.1	206.0–227.0	87.8–96.1
Range (mm)	110.6–228.6	150.4–285.5	57.2–114.4
*p*-value	< 0.001	< 0.001	< 0.001
Difference between 50 and 70-keV (mm)	12.3 ± 1.92	14.4 ± 2.8	4.0 ± 0.9
95% C.I. (mm)	8.5–16.2	8.9–19.9	2.2–5.7
Range (mm)	− 8.9–60.2	− 10.5–69.31	− 3.7–25.6
*p*-value			
Between three	0.001		
RHA versus SMA	0.74		
RHA versus RA	0.01		
SMA versus RA	0.001		

VMI, virtual monoenergetic imaging; 95% C.I., 95% confidence interval; RHA, right hepatic artery; CeA, celiac artery; SMA, superior mesenteric artery; ICA, ileocolic artery; RA, renal artery; ASSA, anterior superior segmental artery

#### Difference in length of each artery at 50 and 70-keV VMI

The differences in the length of each artery between 50-keV and 70-keV VMI were as follows: RHA (from RHA origin to A8 end), 12.3 ± 1.92 mm; SMA (from SMA origin to ICA end), 14.4 ± 2.8 mm; and left RA (from left RA origin to ASSA end), 4.0 ± 0.9 mm. The difference in RHA length was significantly greater than that in RA length (*p* = 0.01). The difference in SMA length was also significantly greater than that in RA length (*p* = 0.001). There were no significant differences with respect to the lengths of the RHA and SMA (*p* = 0.74) (Table 4, Fig. 6).

## Discussion

The quantitative image quality for 50-keV VMI was superior to that for 70-keV VMI with respect to the depiction of the arteries in the abdominal region by PCD-CT. In addition, we found that a 50-keV VMI could depict the RHA, SMA, and RA branches more distally than a 70-keV VMI. We are the first to quantify the length of abdominal arteries and the extent to which peripheral arteries are visible with low-keV VMI. We are confident that these findings will make substantially contribute to the field of vascular imaging. Low-keV VMI on PCD-CT will make a significant contribution to clinical practice because the detailed delineation of abdominal vessels will influence treatment planning for surgery and transarterial chemoembolization [27, 28].

Dillinger et al. examined the depiction of abdominal arteries in the VMI of PCD-CT and found that in the 40–190 keV range, 60-keV exhibited the highest CNR of the vessels [29]. Their results showed that the CNR at 50-keV was lower than that at 70-keV VMI, which is inconsistent with our results. They did not describe the iterative reconstruction method or the strength with which they reconstructed the images. Because we reconstructed the images with the highest QIR level, we may have obtained better noise reduction in the CT images than in their study. Furthermore, their study did not describe a specific scan timing for the arterial phase, which may have been different from that of the early arterial phase in our study. In addition, they used Qr44, a quantitative imaging kernel, whereas we used Bv44, a vascular kernel. In addition, this study used a scanning protocol specific to abdominal artery delineation, which may have influenced the results. Booij et al. compared the CNR of iodine between DECT and PCD-CT using an abdominal phantom [30]. They reported that, at energies below 60-keV, the CNR of PCD-CT was significantly higher than that of DECT. This suggests that PCD-CT may improve the arterial delineation of CTA at a lower keV than DECT. In their phantom experiments, the 50-keV CNR of the PCD-CT was higher than that of the 70-keV VMI. This finding is consistent with the results of our study.

The cadmium telluride (CdTe) sensor used for PCD-CT significantly increases the CNR of iodine compared to the scintillator sensor used in conventional CT for the following reasons. The CdTe sensor converts X-ray photons equally weighted from the low to high keV range, whereas the scintillator sensor converts photons downweighted in the lower keV range, resulting in an insufficient CNR of iodine. The CdTe sensor also does not require separators between cells, unlike the conventional CT scintillator, which reduces the image noise by increasing the dose efficiency of the sensor. In addition, the smaller pixelated anode size of PCD-CT significantly improves small vessel depiction by reducing the partial volume effect, which often degrades the signal intensity at distal locations [31]. Combining these factors may improve arterial visualization at low-keV VMI in PCD-CT. The significantly smaller difference in arterial length between 50 and 70-keV in the RA may be the result of a lower contrast between the renal parenchyma and peripheral arteries within the renal parenchyma on CTA. In the arterial phase, brisk enhancement was observed in the renal cortex [32]. In contrast, the RHA and SMA strongly contrasted with the surroundings on CTA, which may enable the arteries to be visualized more peripherally using 50-keV VMI.

This study has several limitations. First, this was a single-center, retrospective study with a relatively small number of patients. Second, it was not possible to completely blind 50- and 70-keV VMIs. This is because the difference in the contrast of the iodine contrast agent would reveal whether it was a 50- or 70-keV VMI. Third, we did not evaluate different reconstruction kernels, dose settings, tube voltages, or QIR settings. Depending on the setting, the results can be quite different. Fourth, the results indicate that the abdominal arteries can be visualized more peripherally at 50-keV VMI, but we did not evaluate whether this is actually useful in clinical practice. Fifth, this study used SYNAPSE VINCENT for 3D processing and measurement but did not evaluate the use of other workstations. Finally, no comparison was made between the SNR and CNR of the VMI and those of the 120 kVp images in this retrospective study because the 120 kVp images were not stored on the picture archiving and communication system server.

In conclusion, *low-keV VMI improves the SNR and CNR of abdominal arteries.* Moreover, *VR images from a low-keV VMI enable delineation of more peripheral abdominal arteries on PCD-CT.* Further large prospective multicenter studies are needed to clarify whether this conclusion is clinically useful.
